# Insights from genomic studies on the role of sex steroids in the aetiology of endometriosis

**DOI:** 10.1530/RAF-21-0078

**Published:** 2022-04-04

**Authors:** Philippa T K Saunders

**Affiliations:** 1 Centre for Inflammation Research, The University of Edinburgh, Edinburgh, UK

**Keywords:** women’s health, endometriosis, genome-wide association studies (GWAS), single nucleotide polymorphism (SNP), oestrogens, androgens

## Abstract

Endometriosis is a chronic neuro-inflammatory disorder the defining feature of which is the growth of tissue (lesions) that resembles the endometrium outside the uterus. Estimates of prevalence quote rates of ~10% of women of reproductive age, equating to at least 190 million women world-wide. Genetic, hormonal and immunological factors have all been proposed as contributing to risk factors associated with the development of lesions. Twin studies report the heritable component of endometriosis as ~50%. Genome-wide association studies (GWAS) have identified single nucleotide polymorphisms (SNPs) that appear over-represented in patients with endometriosis, particularly those with more extensive disease (stage III/IV). In different sample populations, there has been replication of SNPs near genes involved in oestrogen and other steroid regulated pathways including *ESR1* (oestrogen receptor alpha), *GREB1*, *HOXA10*, *WNT4* and MAPK kinase signalling. Comparisons with GWAS conducted on other patient cohorts have found links with reproductive traits (age at menarche) and disorders (fibroids, endometrial and ovarian cancer) and common co-morbidities (migraine, depression, asthma). In summary, genetic analyses have provided new insights into the hormone-regulated pathways that may contribute to increased risk of developing endometriosis some of which may act in early life. New studies are needed to clarify the relationship between the many SNPs identified, the genes that they regulate and their contribution(s) to development of different forms of endometriosis. We hope that more advanced methods allowing integration between GWAS, epigenetic and tissue expression data will improve risk analysis and reduce diagnositic delay.

**Lay summary:**

Endometriosis is a debilitating reproductive disorder affecting ~10% of reproductive-age women, and those assigned female at birth, which causes a range of symptoms including chronic pain and infertility. The reason why some, but not all these individuals, develop the lesions that characterise the disease are poorly understood, but recently attention has focused on genetic risk factors to explain why the incidence is higher in some families. Studies on large cohorts of patients with comparison of their DNA to women without endometriosis or with other disorders have documented changes in genes associated with steroid hormone production or action. The results provide further evidence that endometriosis shares genetic risk factors with other disorders of the reproductive system and a platform for new ideas related to risk, biomarkers and therapies.

## Introduction

Endometriosis is a complex, heterogeneous, chronic, incurable disorder the hallmark of which is growth of tissue ‘lesions’ that have histological features resembling the intrauterine (eutopic) endometrium outside the uterine cavity (Horne & Saunders 2019, Zondervan *et al.* 2020). Estimates of prevalence typically quote rates of ~10% of women of reproductive age, equating to 190 million individuals world-wide (Zondervan *et al.* 2020). This is likely to be an underestimate as many women, or those assigned as female at birth, may remain undiagnosed, and ‘lesions’ have also been found in asymptomatic fertile women (Shafrir *et al.* 2018). Prevalence rates can be as high as 50% in women seeking treatment for infertility (Meuleman *et al.* 2009): rates in adolescents with pelvic pain range from 49% to 75% (Shafrir *et al.* 2018). A recent review highlighted the profound negative impact on the lives of individuals with the disorder (Missmer *et al.* 2021).

**Figure 1:**
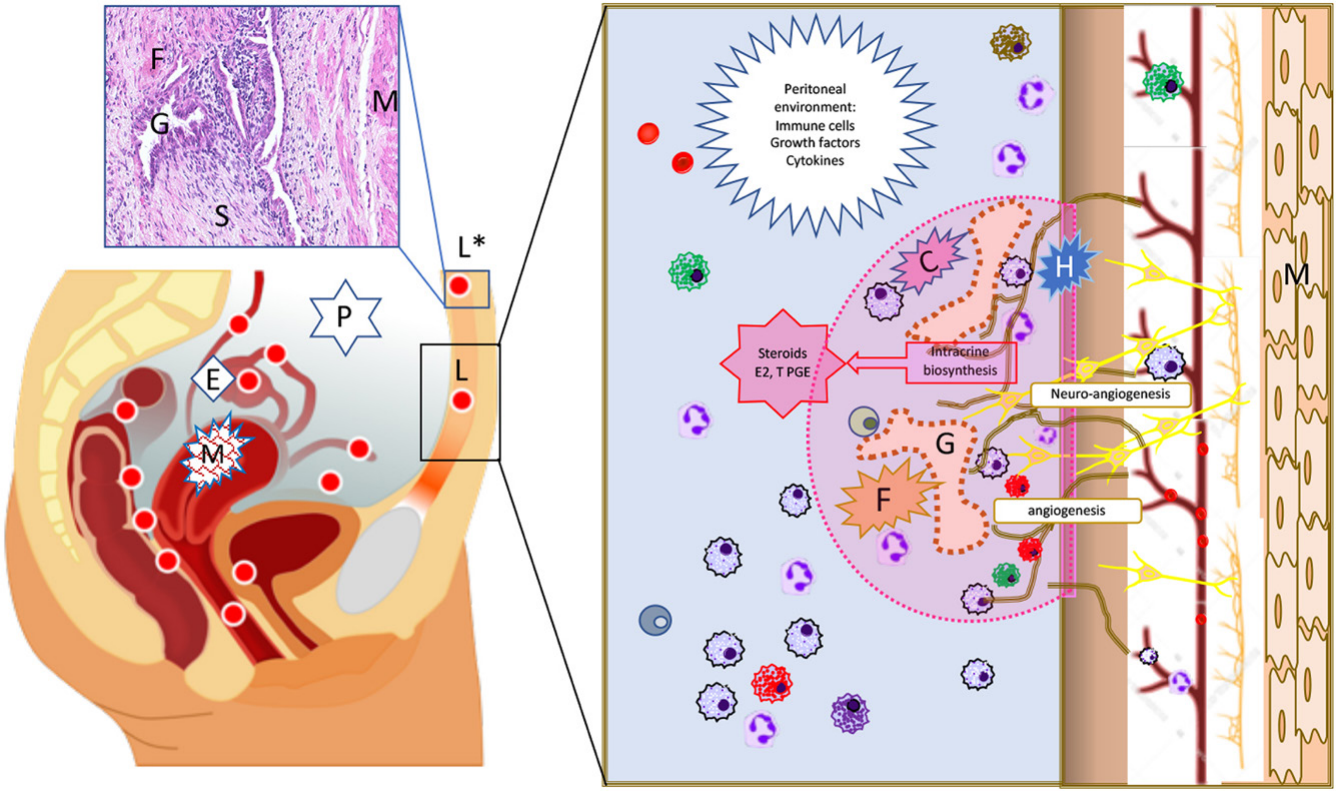
Location and histology of endometriosis lesions. Endometriosis lesions are predominantly found in the pelvic cavity where they may be associated with the peritoneal wall (superficial peritoneal), the ovary (cysts/endometriomas) or as nodules (deep endometriosis) associated with areas of fibrosis and adhesions between bowel, bladder and vagina (lower left diagram shown as red circles). A histological section of a superficial peritoneal lesion (*) stained with H&E is shown above the diagram of the pelvic cavity. The lesion is supported by the peritoneal wall which has a layer of smooth muscle (M), and it contains stromal fibroblasts (S), myofibroblasts (fibrosis, F) and a gland surrounded by epithelial cells (G). The right-hand panel shows a diagrammatic representation of a lesion (surrounded by dotted pink line) mirroring the histology of the H&E image complemented by representation of additional cell types including nerves (yellow), blood vessels (brown) and immune cells (variety of colours). It also shows processes that contribute to lesion survival (angiogenesis), growth of nerves in lesions (neuroangiogenesis) and creation of a unique environment that has high concentrations of steroids (intracrine biosynthesis).

Endometriosis lesions are most commonly found within the pelvic cavity (Fig. 1) (Zondervan *et al.* 2020) but may also occur in other sites including the thorax and nervous system (Andres *et al.* 2020). The location, type, degree of invasion, extent of disease and associated adhesions have been used to ‘stage’ the disease with the most widely adopted scheme being that proposed by the American Society of Reproductive Medicine. This scheme proposes classification of endometriosis lesions into four stages I to IV: stage I – mild, stage II – minimal, stage III – moderate, stage IV – severe. The assignment of stage is based on visual analysis at time of surgery and a points-based system with the majority of peritoneal disease scored as stage I/II and more extensive disease associated with adhesions and deep nodules as stage III or IV.

Endometriosis is associated with a wide range of symptoms including pelvic pain (which may be more severe during menstruation), painful sex, heavy menstrual bleeding, bladder and bowel symptoms as well as those shared with other chronic pain conditions such as fatigue and depression. Several reports have recorded poor correlation between patient-reported pain and endometriosis stage (Vercellini *et al.* 2007). Some commentators have suggested endometriosis should be considered a ‘syndrome’ with greater emphasis on symptoms rather than lesion subtype/location (Saunders & Horne 2021). Results from genomic and other studies (discussed below) suggest there may also be differences in the aetiology of deep and ovarian disease compared with peritoneal, superficial lesions.

In this narrative review, I will consider the evidence that genetic changes in gene pathways associated with hormone production or action may contribute to the risk of endometriosis and other disorders that may have shared genetic risk factors. Opportunities to use the information to improve non-surgical diagnosis or personalised therapies will be briefly discussed.

## Why is endometriosis usually referred to as a ‘hormone-dependent disorder’?

Endometriosis only occurs spontaneously in menstruating species including humans and some primates. Explanations for the formation of endometriosis lesions, particularly those in the pelvis, have largely focused on a theory, first proposed in the 1920s, that tissue fragments including stem/progenitor cells and immune cells transferred via fallopian tubes at the time of menstruation (retrograde flow) survive and become attached to the peritoneal wall and other sites (Horne & Saunders 2019, Zondervan *et al.* 2020). Other theories include transfer via the vasculature and (Yovich *et al.* 2020) and coelomic metaplasia (Zondervan *et al.* 2020).

The human endometrium is a complex multicellular tissue that is exquisitely sensitive to the actions of sex steroids with cycles of proliferation, differentiation breakdown, shedding (menstruation) and repair orchestrated by changes in circulating concentrations of endocrine hormones secreted by the ovaries (Gibson *et al.* 2020). Within the ovaries, follicular maturation, and subsequent expression of steroid enzymes such as aromatase (metabolises androgens to oestrogens) within the granulosa cells (Turner *et al.* 2002), is stimulated by the pituitary hormones LH and FSH. G-protein coupled receptors that bind FSH (FSHR) are expressed in the granulosa cells, and there are reports that FSHR polymorphisms are associated with increased risk of endometriosis in fertile women (Andre *et al.* 2018).

The actions of sex steroids, including oestrogens, progestins and androgens, are mediated by receptors encoded by genes (*ESR1, ESR2, PR, AR*respectively) that are members of a large family of ligand-activated transcription factors. Studies using cell-based assays and mouse models have highlighted a key role for ESR1 (oestrogen receptor alpha) in regulation of endometrial cell proliferation and stromal–epithelial cell signalling (Winuthayanon *et al.* 2017). Progesterone acting via PR, in combination with other factors including cAMP, plays a critical role in the differentiation (decidualisation) of endometrial stromal fibroblasts resulting in remodelling of their cytoskeleton (shape), changes in gene expression and a novel secretory profile (Gellersen & Brosens 2003). Decidualisation is associated with increased synthesis and secretion of factors such as interleukin 15 that regulate recruitment of immune cells including the CD56+ uterine natural killer cells (uNK) which play a key role in remodelling of the vasculature (Gibson *et al.* 2015). Androgens and AR also play an important role in regulation of endometrial function and endometrial pathologies (Gibson *et al.* 2020) with strong expression of AR in stromal fibroblasts and variable expression in epithelial cells in normal endometrium. Steroid metabolism within lesions results in high levels of testosterone which are independent of stage of the cycle (Huhtinen *et al.* 2014). Expression of AR and 5 alpha reductase enzymes capable of metabolising testosterone to the highly potent steroid dihydrotestosterone in lesions has also been reported (Carneiro *et al.* 2008).

Studies on the role of steroids in the pathogenesis of endometriosis have included evidence of changes in the function of the endometrium in women with endometriosis, measurement of steroids in the peritoneal fluid and detailed analysis of expression of steroid receptors, metabolising enzymes and steroid concentrations in the lesions themselves (Fig. 1). The results of these studies have consistently reported differences between samples from women with or without endometriosis that involve steroid hormone production or action including altered responses to progesterone (‘progesterone resistance’) (Burney *et al.* 2007). There is also a large body of work that has identified changes in expression of enzymes and the creation of an oestrogen-dominated microenvironment within the lesions which has provided the rationale for the use of enzyme inhibitors, including those targeting the aromatase enzyme, as therapeutics (Dunselman *et al.* 2014).

Readers interested in learning more about the many studies exploring the role of steroid receptors in endometriosis are encouraged to read the comprehensive review by Yilmaz and Bulun that summarises papers published up to 2018 (Yilmaz & Bulun 2019).

## Evidence that endometriosis is a heritable disease

Patients often report cases of endometriosis in close relatives. A study using questionnaires explored the incidence of endometriosis in female monozygotic and dizygotic twins in the Australian National Health and Medical Research Council Twin Register. More than 3000 twins responded with 215 twins recording a diagnosis of endometriosis; when available medical and pathology reports were included, the authors concluded ~51% of the variance of the latent liability to endometriosis could be due to additive genetic influences (Treloar *et al.* 1999). In a subsequent study, the same group published a key paper based on genetic linkage analysis of 1176 families in Australia and the UK with at least 2 affected individuals (Treloar *et al.* 2005). They used a positional-cloning approach that starts with linkage analysis to identify genomic regions likely to harbour genes that contributed to disease predisposition. They identified significant linkage (MLS = 3.16) to a novel susceptibility locus on chromosome 10q26. Notably, a subsequent GWAS study (described below) identified a number of genetic polymorphisms in this region associated with endometriosis using larger numbers of patient samples (Painter *et al.* 2011*a*). A recent study used population data in Korea to quantify the familial risk of endometriosis among full siblings (19,195 women with 1126 cases) to examine interactions between family history, smoking, age at menarche and BMI (Kim *et al.* 2021). This study endorsed the findings of earlier studies showing increased risk associated with having an affected sibling which was higher in twins.

## Genomic studies on samples from women with and without a diagnosis of endometriosis have identified gene polymorphisms that appear associated with disease risk

A large number of studies have focused on specific changes in gene expression in lesions and/or endometrium from women with endometriosis (Table 1; reviewed by Yilmaz & Bulun 2019, Zondervan *et al.* 2020, Saunders & Horne 2021). To complement these data, other investigations have looked for polymorphisms in steroid receptor genes. For example, Kitawaki *et al.* examined the distribution of PVUII genotypes (PP, Pp and pp) in *ESR1* using DNA from blood samples (203 women, 109 with a diagnosis of endometriosis) reaching the conclusion that the Pp/pp variants were higher in women with endometriosis, fibroids or adenomyosis (Kitawaki *et al.* 2001). Based on analysis of 72 women with deep endometrial disease, van Kaam and colleagues reported the presence of the PR gene polymorphic allele +331A was associated with reduced risk of endometriosis compared to a healthy population (*n* = 102) (van Kaam *et al.* 2007). Negative findings in other studies are in part believed to be due to low numbers of patients versus controls something that has now been addressed using large-scale unbiased genome scanning (genome-wide association studies, GWAS). In support of this approach, Zondervan and colleagues highlighted the technical developments including the generation of data in the 1000 genomes project and improved statistical analysis that have made GWAS analysis more robust and a good platform for unbiased analysis of genetic changes (Zondervan *et al.* 2016). For complex diseases such as endometriosis, where changes in the levels of expression of individual gene may only make a small overall contribution to the aetiology of the disorder, very large numbers of individuals need to be evaluated. To ensure that valid comparisons are made it is also important that the endometriosis phenotype of patients has been recorded using robust clinical criteria and controls are drawn from populations with identical ancestry. A summary of key GWAS conducted on endometriosis patients (including stage of disease where this is known) is given in Table 2, and the results that appear to implicate steroid regulated pathways in the development of the disease are discussed in more detail below.

**Table 1:** Summary of key studies that have provided evidence for altered steroid biosynthesis and/or action in eutopic endometrium or endometriosis lesions from patients.

Title/topic	Methods	Results	References
Expression of ESR1 vs ESR2 in endometriosis lesions compared with endometrium	Fibroblasts isolated from endometrioma and endometrium, immunohistochemistry, evaluation of methylation status of *ESR2* promoter	ESR2 overexpressed in lesions compared with ESR1; mechanism involving altered methylation	Xue *et al.* (2007)
Gene expression analysis of endometrium reveals progesterone resistance and candidate susceptibility genes in women with endometriosis	Endometrial tissue biopsies from 21 women with endometriosis and 16 women without, cycle stage determined. Affymetrix arrays+gene ontogeny	Phase-dependent changes in gene expression in both tissue sets. Patient samples – enrichment of genes involved in proliferation in early secretory phase dysregulation of P target genes in secretory phase	Burney *et al.* (2007)
Gene expression profile for ectopic vs eutopic endometrium provides new insights into endometriosis oncogenic potential	Paired samples of endometriomas and endometrium (12 women, luteal phase). Nimgen microarrays, validation of 20 genes, pathway analysis (DAVID)	Cluster-dependent modulation of HOX genes Altered cell cycle genes (suppressed?)	Borghese *et al.* (2008)
Prostaglandin E2 via SF-1 coordinately regulates transcription of steroidogenic genes necessary for oestrogen synthesis in endometriosis	Stromal cells isolated from wall of endometriomas (17) and endometrium (16), extra-ovarian tissue from **different**group of women (13). RT-PCR steroid enzyme mRNAs, ChIP assays	StAR, P450scc, P450c17, P450arom are higher in ectopic samples. SF1 is high and binds promoter of StAR	Yilmaz and Bulun (2019)
Intra-tissue steroid profiling and enzyme analysis confirms differences in steroid metabolism in the endometrium and endometriosis lesions	RT-PCR analysis of enzymes in lesions vs endometrium, LC-MS/MS for direct measurement of steroid concentrations	Altered tissue steroid concentrations in endometriosis; altered expression of various steroidogenic enzymes HSD3B2 high CYP11A1 low	Huhtinen *et al.* (2012)

**Table 2:** Larger GWAS studies that have identified common SNP variants associated with endometriosis.

Patients	Controls	SNPs	Genes/pathways	Reference
3194 surgically confirmed	Population controls 7060 (UK/Australia)	7p15.2 strongest in subgroup with stage III/IV disease; rs12700667	Intergenic region upstream of *NFE2L3, HOXA10/A11*	Painter *et al.* (2011*a*)
3223 women with surgically confirmed endometriosis, 1090 women without endometriosis	Population controls 7060	Examined 11,984 SNPs on chromosome 10. Signal 10q26 rs11592737 replicated	*CYP2C19*	Painter *et al.* (2011*b*)
4604 women with surgically confirmed endometriosis	9393 women of Japanese and European ancestry	rs12700667 replicated in Japanese; rs7521902 at 1p36.12; rs13394619 at 2p25	*NFE2L3, HOXA10* *WNT4* *GREB1*	Nyholt *et al.* (2012)
Meta-analysis of 11,506 cases: stage III/IV 2859	32,678 (European and Japanese)	8 of 9 loci have stronger effect in stage II/IV; rs1537377; rs13394619 rs12700667; rs7521902; rs7739264	*CDKN2B-AS1; GREB1; WNT4; VEZT; ID4*	Rahmioglu *et al.* (2014)
3908 diagnosis of endometriosis	8568 women of Japanese and European ancestry	rs6542095, rs3783550 rs3783525	IL4 locus	Sapkota *et al.* (2015*b*)
2594 Australians with positive diagnosis	4496 controls	rs3820282; rs12038474; + blood eQTL	Interactions with promoters of *LINC00339*, *CDC42* (silencer)	Powell *et al.* (2016)
3194 (surgical confirmed) stage I/II 1686; stage III/IV 1364	7060 controls European ancestry	rs144240142 (intronic MAP3K4)	MAPK signalling pathway; ECM glycoprotein	Uimari *et al.* (2017)
Metanalysis 17,045 cases	191,596 controls	FN1, CCDC170, ESR1, SYNE1 and FSHB	Steroid receptors and steroid signalling	Sapkota *et al.* (2017)
Pooled GWAS, endometrioma, Han Chinese women 50 primary and 1448 for validation	1540 (had secondary infertility or fibroids)	10 novel loci	Most significant: IGF1R (signalling); Meis homeobox	Wang *et al.* (2017)

In their 2011 studies, Painter and colleagues identified SNPs on 7p15.2 (Painter *et al.* 2011*a*) and 10q26 (Painter *et al.* 2011*b*) in regions of the genome that appeared associated with *HOXA10* and *CYP2C19C*. The finding of a SNP associated with *HOXA10* was exciting as there is a large body of work that has highlighted the importance of HOX genes in development of the female reproductive tract and differentiation of the endometrium in preparation for pregnancy discussed below (Du & Taylor 2015).

In a follow-up study, the authors re-analysed 80 SNPs highlighting rs4244285, a functional SNP in exon 5 of* CYP2C19,*that abrogates its function through the creation of an alternative splice site and another functional SNP in the* CYP2C19*promoter. The authors proposed that variants of *CYP2C19* may contribute to endometriosis susceptibility in both familial and sporadic cases (Painter *et al.* 2014). CYP2C19 is a member of the cytochrome P450 enzyme superfamily often implicated in drug metabolism by the liver. In the context of endometriosis, it is notable that this enzyme acts as an expoxygenase that can convert arachidonic acid to four epoxyeicosatrienoic acid regioisomers which have diverse impacts on blood vessels and inflammation (Spector 2009). Whilst there is no specific evidence for a role for CYP2C19 in endometrium or endometriosis, it is notable that expoxygenase activity has been studied in the context of macrophage activity in wounding and fibrosis both processes relevant to development of endometriosis lesions (Guo 2018).

Several primary GWAS and subsequent meta-analyses have reported associations between endometriosis and SNPs near genes involved in signalling pathways implicated in endometrial tissue function (Table 2). For example, Nyholt *et al.* (2012) identified rs7521902 at 1p36.12 near *WNT4*; thereafter, the group conducted fine mapping of 1p36 region spanning *WNT4, CDC42* and *LINC00339* finding three additional SNPs located in DNA sequences with potential overlap with binding sites for *FOXA1, FOXA2, ESR1* and *ESR2* (Luong *et al.* 2013). In their meta-analyses, which incorporated data from eight of the GWAS conducted before 2014, Rahmiglou and co-investigators confirmed significance for SNPs associated with *WNT4, CREB1* and *VEZT*. They also highlighted the stronger effect sizes among women diagnosed with more extensive or ovarian disease (stage III/IV) for eight of the SNPs (Rahmioglu *et al.* 2014). Other studies have also reported that the most robust findings are found if results are stratified and sorted according to disease stage (Sapkota *et al.* 2015*a*).

A meta-analysis with more than 17,000 patients and 191,000 controls identified 5 novel SNPs associated with steroid signalling pathways as well as 5 secondary association signals, including two at the *ESR1* locus, resulting in 19 independent SNPs which the authors postulated might contribute to 5.19% of variance in endometriosis (Sapkota *et al.* 2017). Whilst these studies have revealed some promising leads, the population studied has largely been limited to women with European ancestry although the 7p15.2 SNP was also replicated in Japanese women (Nyholt *et al.* 2012). In a small study Wang *et al*. focused on endometriomas in Han Chinese women finding the most significant signalling pathway was that associated with IGF1 receptor (Wang *et al.* 2017) which is interesting as macrophage-derived IGF1 has recently been highlighted as a nerve sensing factor in endometriosis-associated pain (Forster *et al.* 2019).

Replication and meta-analysis of previous GWAS confirmed vezatin as a locus having a strong association with endometriosis in Italian patients (Pagliardini *et al.* 2015). Immunostaining of endometrium suggested the protein was in multiple cell types and not altered according to cycle stage; in the same study, the authors focused on the 12q22 region and explored whether the SNPs found in this region that are associated with the *VEZT* gene (rs10859871) had an impact on expression in endometrial tissue samples (eQTL analysis) (Holdsworth-Carson *et al.* 2016). A total of 11 coding variants of *VEZT*(including 1 novel variant) were identified from an endometriosis cohort consisting of 2594 cases and 4496 controls, but they did not find any definitive evidence of a change in VEZT protein expression in subset of endometrial tissue samples (*n* = 122) concluding further validation was needed of a relationship between SNP and gene expression levels.

Using* in vitro* approaches and blood eQTL analysis, a SNP at rs12038474 was found to be located in transcriptional silencer for *CDC42* and to increase its expression in reporter assays (Powell *et al.* 2016). CDC42 is a member of the Rho family of GTPase signalling molecules, and its overexpression in some cancers has been implicated in increased cell migration. Other studies have reported that stromal and stem cells from women with endometriosis have an altered phenotype associated with enhanced migration and suggested this may involve Rho/ROCK signalling pathways (Yotova *et al.* 2011). Therapies targeting CDC42-dependent pathways are being explored as treatments for cancers with high mortality such as non-small cell lung cancer (Tan *et al.* 2020). Expression of CDC42 was previously investigated using immunostaining of eutopic and ectopic endometrium in 19 patients with ovarian endometriosis (Goteri *et al.* 2006) with some weak evidence of increased expression in secretory phase endometrium in those with disease.

## Evidence from genomic studies supporting a role for sex steroids in the aetiology of endometriosis

Evidence from GWAS (Table 2) appear consistent with a role for genetic mutations in genes implicated in steroid regulation of the endometrium in modifying the risk of developing endometriosis. Follow-up studies, reviewed briefly below, have been conducted which have further strengthened this evidence and complemented studies on individual hormone-dependent gene expression in endometrial cells and tissues (Burney *et al.* 2007).

In a meta-analysis, Nyholt and collaborators used data from 11 GWAS case–control data sets with more than 17,000 endometriosis cases (Table 2) (Sapkota *et al.* 2017). They replicated previous reported loci and identified five novel SNPs significantly associated with genes involved in sex steroid hormone signalling pathways including FSH beta (*FSHB*), fibronectin (*FN1*) and CCDC170 a gene implicated in breast cancer risk (Dunning *et al.* 2016) and five secondary association signals, including two at the *ESR1* locus. Given the importance of FSH in regulating production of oestrogens by the ovarian granulosa cells, the finding of SNPs associated with the *FSHB* gene provides a further link between endometriosis risk and oestrogen action(s). Notably in their paper, Sapkota *et al.* (2017) reported that their data were supported by independent samples from the UK Biobank (Ruth *et al.* 2016) and the index SNP was in high linkage disequilibrium with other SNPs associated with FSH concentrations.

SNPs associated with the *ESR1* gene and *GREB1*, an early response gene that is regulated by oestrogens as well as androgens in hormone-dependent cancers (Cheng *et al.* 2018), were reported in endometriosis GWAS including large-scale meta-analysis (Rahmioglu *et al.* 2014, Sapkota *et al.* 2017), although not all studies have replicated findings of an association with the rs11674184 SNP of the *GREB1*gene (Matalliotaki *et al.* 2019). Oestrogen receptors play a key role in regulation of endometrial function, and it is notable that many studies have recorded dysregulation of *ESR1/ESR2* with overexpression of the latter in endometriosis lesions (Table 1) which has been attributed to changes in methylation status of *ESR2* (Xue *et al.* 2007) rather than genomic SNPs.

In a recent paper, Marla *et al.* (2021) examined hormonal and genetic regulation of genes in the *ESR1* region in endometrium and explored the effect of endometriosis risk variants. The authors noted that variants in the *ESR1* region of SNPs associated with endometriosis risk were not the same as the *ESR1* SNPs associated with age at first birth, age at menarche or breast cancer which is something that needs to be born in mind when linking risks to pathways.

SNPs associated with *HOXA10* (7p15.2) have been reported in more than one GWAS (Painter *et al.* 2011*a*, Nyholt *et al.* 2012). Expression of HOXA10 is steroid regulated in adult endometrium: two EREs that can bind either ESR1 or ESR2 *in vitro* have been identified in the regulatory region of the gene highlighting a direct link to oestrogens and oestrogen receptor action in endometrial cells (Akbas *et al.* 2004). The abundance of HOXA10 protein in endometrial stromal cells increases as they decidualise in response to progesterone and it plays a key role in regulating other genes implicated in regulation of metabolism, DNA replication and repair, cell junction, and lysosome and signal transduction (Wang *et al.* 2021). Miss-expression of HOXA10 has been reported to contribute to infertility (Ashary *et al.* 2020) and mice with deletion of *Hoxa10* have severe defects in decidualisation and implantation (Gao *et al.* 2015). More recent studies have suggested altered expression of HOXA10 might also be a risk factor for adenomyosis (abnormal invasion of endometrium into the myometrium) which is often found as co-morbidity with endometriosis consistent with these conditions sharing common risk factors (Bulun *et al.* 2021).

Members of the Wnt gene family are well established as regulators of endometrial cell function with important roles in epithelial–mesenchymal interactions (Tulac *et al.* 2003). SNPs associated with *WNT4* (at 1p36.12) have been reported in several GWAS (Rahmioglu *et al.* 2014). This gene encodes a secreted signalling factor that regulates both development of endometrial glands and progesterone signalling during decidualisation (Franco *et al.* 2011, Hayashi *et al.* 2011). In a study comparing expression of WNT4 in eutopic and ectopic endometrium of 30 patients with endometrium from 30 controls, some evidence was presented for downregulation in ectopic endometrium and in eutopic endometrium of patients compared with controls (Liang *et al.* 2016) although more extensive studies to link SNPs to gene expression are required.

Whilst the evidence described above appears to add weight to the link between genetic changes in regions of the genome that appear to be associated with genes involved in steroid regulation of endometrium/endometriosis lesions, it is important to acknowledge that steroids can have pleiotrophic effects that span development as well as adult life making cause and effect difficult to unravel particularly in a disorder with a complex presentation. Much larger-scale studies are now required to expand on the link between SNPs and the regulation of gene expression that is specific to endometrium (Mortlock *et al.* 2020).

## Genomic studies have revealed links between endometriosis reproductive traits and other disorders

The rapid increase in large-scale GWAS has opened up the opportunity to compare SNPs in women with endometriosis with those identified as associated with reproductive traits and reproductive or other disorders (Table 3).

**Table 3:** GWAS studies from reproductive and other disorders that have identified SNPs in common with endometriosis.

Condition	Cohort for non-endo condition	SNP overlap with endometriosis	Target genes/pathways	References
Fibroids	35,474 cases and 267,505 female controls of European ancestry	1p36.12, rs7412010; 2p25.1, rs35417544; 6q25.2, rs58415480; 11p14.1, rs11031006	WNT4, CDC42, GREB1, ESR1, FSHB	Gallagher *et al.* (2019)
Age at menarche	395 patients (endo), 981 controls	52 SNPs previously identified for age at menarche: 16 SNPs overlap with endo; rs6589964	28 genes in G alpha signalling pathway; *LHCGR*several SNPs (strong); *BSX*– increases affinity for FOXA transcription factors	Ponomarenko *et al.* (2020)
Endometrial cancer	4 data sets: 6459 patients, 32,624 controls	13 loci incl rs2475335 located in PTPRD	STAT3 pathway	Painter *et al.* (2018)
Endometrial cancer	Data from O’Mara *et al.* (2018) with replication using UK Biobank 12,270 cases/46,126 controls	4 regions identified with 17q21.32 demonstrating evidence of a shared genetic risk signal; 3q21.3? novel	Potential genes?: CBX1, MIR1203, SKAP1 SNX11	Kho *et al.* (2021)
Ovarian cancer	10,065 cases and 21,663 controls	Clear cell carcinoma showed the strongest genetic correlation with endometriosis	??	Lu *et al.* (2015)
Obesity/leanness	BMI (GIANT; 123,865 individuals) and WHRadjBMI (GIANT: 77,167 individuals)	7p15.2; KIFAP3 and CAB39L are novel associations for both traits	Wnt pathway (3 genes)	Rahmioglu *et al.* (2015)
Migraine	22 GWAS, 59,674 migraine cases and 316,078 controls (sex considered as a covariant)	SNPs near *SLC35G6, TRIM32, ARL14EP*	IL1R binding, PI3K-Akt-mTOR-signalling, MAPK signalling, TNF-α signalling	Adewuyi *et al.* (2020)
Depression	170,756 cases of depression 329,443 controls of European ancestry	20 independent loci, 8 novel	Causal relationship?; Gastric mucosal abnormality	Adewuyi *et al.* (2021)
Asthma	UK Biobank 26,332 cases of asthma/ 375,505 controls; TAGC consortium 19,954 cases/107715 controls	UKB comparison 14 independent loci, 5 putative novel (3 replicated in TAGC)	Biological pathways including thyroid hormone signalling, androgen biosynthetic process	Adewuyi *et al.* (2022)

Younger age at menarche has been implicated in increased risk of developing endometriosis as having short menstrual cycles and low BMI whereas having more children is associated with lower risk (Shafrir *et al.* 2018). GWAS studies have shed light on the heritable factors that may contribute to these characteristics with comparisons made to endometriosis data sets. For example, a large-scale GWAS has identified a genetic component to age at first birth and number of children with 12 loci including an SNP associated with *ESR1*(rs4851269) (Barban *et al.* 2016). There is also evidence from GWAS studies for a shared genetic risk factors between ovarian ageing and premature ovarian failure (McGrath *et al.* 2021). A preprint article (https://doi.org/10.1101/401448) which has not yet been peer reviewed reported a GWAS of endometriosis-related infertility, including 2969 cases and 3770 controls; they did not show genome-wide significance for any SNPs associated with endometriosis-related infertility although they recorded three SNPs at or near genes implicated in female fertility in model organisms.

To identify loci for age at menarche, a meta-analysis of 32 GWAS in 87,802 women of European descent, with replication in up to 14,731 women, was performed resulting in the identification of more than 30 new SNP loci (Elks *et al.* 2010). Notably three of these were in or near genes implicated in hormonal regulation (*INHBA, PCSK2, RXRG*). A more recent small-scale study took 52 of the candidate SNPs for age at menarche and their gene–gene and gene–environment interactions and analysed whether they were associated with endometriosis using samples from 395 patients and 981 controls (Ponomarenko *et al.* 2020). They found 16 SNPs that were associated with endometriosis and evidence for a link with the G protein signalling pathway. One of the most well-established associations with age at menarche is body size with early studies indicating this is regulated by genetic factors rather than diet (Stark *et al.* 1989). It is therefore of note that Rahmioglu and colleagues have reported a significant enrichment of common SNPs when comparing data sets based on fat distribution and endometriosis (Rahmioglu *et al.* 2015) including shared genes associated with the WNT signalling pathway (Table 3). A recent analysis using two-sample randomization also found evidence that reduced body weight/BMI and variants that expose women to more episodes of menstruation might be mediating genetic susceptibility to endometriosis (Garitazelaia *et al.* 2021) which backs up epidemiological and other genetic data including GWAS discussed above.

Comparisons have made between SNP data from endometriosis patients and those from women with fibroids (leiomyomata) (Gallagher *et al.* 2019). A meta-analysis reported that genes associated with endometriosis that were involved in hormone signalling (*WNT4/CDC42, GREB1, ESR1, FSHB*) were also associated with diagnosis of fibroids. The authors reported that there was at least a doubling of risk for a diagnosis of fibroids among those with a history of endometriosis suggesting overlapping genetic origins. Notably, candidate genes identified for age at menarche are also associated with presence of fibroids. A recent study reported that of the 23 loci associated with fibroids, 16 were associated with either age at menarche (7 SNPs) or height and/or BMI (13 SNPs) (Ponomarenko *et al.* 2020). One of the SNPs was associated with at least two of the three phenotypes being rs4374421 (associated with *LHCGR)*consistent with an important role for hormones/receptors in regulation of multiple reproductive phenotypes.

Epidemiological and array studies have identified an increased risk of developing some forms of ovarian cancer in women with endometriosis (Lu *et al.* 2015). Analysis of endometriosis and endometrial cancer SNP data sets (Painter *et al.* 2018) highlighted 13 distinct loci associated with both endometriosis and endometrial cancer. The study suggested that endometriosis and endometrial cancer have a moderate, but significant, shared genetic aetiology. Recently, Japanese researchers performed GWAS studies of two benign gynaecologic diseases (endometriosis, fibroids) and three reproductive cancers (ovarian, endometrial and cervical) using data of 46,837 subjects and 39,556 matched female controls from the Japan Biobank Project (Masuda *et al.* 2020). They reported genetic correlations were relatively strong between ovarian cancer and endometriosis and reported a weaker association between endometriosis and fibroids as well as SNPs in endometrial and ovarian cancer unique to Japanese and/or East Asians. In a recent study, Australian researchers also identified genetic risk regions shared between endometriosis, endometrial cancer and fibroids and a novel genome-wide significant endometrial cancer risk locus at 1p36.12, contained biologically relevant genes, including *WNT4* discussed above (Kho *et al.* 2021) (Table 3). In this study, the authors used a GWAS data set from endometrial cancers and an expanded data set of 12,906 cases to identify 9 new SNPs with complementary analysis of epigenomic marks in cell lines showing greater overlap in oestrogen-treated cells (O’Mara *et al.* 2018). Comparison with the endometriosis SNP data is available in a preprint (bioRxiv. https://doi.org/10.1101/406967). Whilst stringent analysis using replication data sets failed to replicate some earlier data, the authors did report finding four shared genetic risk regions, three of which (9p21.3, 15q15.1 and 17q21.32) have previously been independently associated with risk of both diseases (Sapkota *et al.* 2017, O’Mara *et al.* 2018).

The finding of common SNPs between endometriosis and migraine (Adewuyi *et al.* 2020) is interesting because they align with reports that migraine is more common in women than men and many women report worse symptoms during menstruation suggestive of an impact of hormones. The co-morbidity of endometriosis with migraine has been reported in a number of epidemiological studies (Yang *et al.* 2012). Notably, in a twin-based study of 815 monozygotic and 457 dizygotic female twin pairs, Nyholt and colleagues reported a significant additive genetic correlation and bivariate heritability between migraine and endometriosis (Nyholt *et al.* 2009). Meta-analysis of endometriosis and migraine GWAS data sets did not find novel genome-wide significant SNPs nor evidence of a causal link however they did identify some SNPs associated with genetically controlled biological mechanisms which might explain the co-occurrence of the two disorders. These included several signalling pathways previously noted in GWAS studies on endometriosis such as IL1R, MAP kinase and Akt-mTOR (Adewuyi *et al.* 2020).

Depression and fatigue are symptoms commonly reported by women with endometriosis (Saunders & Horne 2021). A meta-analysis of endometriosis and depression GWAS (sample size 709,111) identified 20 independent genome-wide significant loci of which 8 were novel (Adewuyi *et al.* 2021). Genes overlapping the two traits were significantly enriched for the biological pathways ‘cell-cell adhesion’, ‘inositol phosphate metabolism’, ‘Hippo-Merlin signalling dysregulation’ and ‘gastric mucosa abnormality’.

New data highlighting shared genetic traits with asthma also align with the strong association between endometriosis and inflammatory processes (Adewuyi *et al.* 2022) (Table 3). Notably in their paper the authors highlighted the many lines of evidence that exposure to high levels of oestrogens increases the risk of asthma (Keselman & Heller 2015, Keselman *et al.* 2017) providing a plausible biological link between the two conditions.

## Have genomic studies provided any new diagnostic or therapeutic opportunities?

Genetic changes identified by GWAS or other methods based on sequencing of DNA arise in the germline, and their impact may therefore be at any time during formation, differentiation or function of a differentiated tissue. The results from these approaches need to be complemented by analysis of cells recovered from lesions or the endometrium of women with endometriosis that can provide information on somatic mutations, epigenetic changes and transcriptomes. One of the main reasons genetic studies were carried out was in anticipation they might lead to the development of screening panels for genes implicated in endometriosis reducing the need for surgical diagnosis. A recent study on Korean women which explored familial cases of endometriosis found shared risk factors/SNPs suggested women with an affected sibling, early menarche, low BMI or who smoked could be considered an at-risk population (Kim *et al.* 2021). This study shows the power of combining information from several studies to move the field forward towards the goal of personalised risk assessment. Notably this study was conducted in Asia whereas nearly all the other GWAS have largely focused on populations with European ancestry: there is clearly an urgency to increase the ethnic diversity of populations studied in GWAS for all reproductive traits and disorders. Another notable limitation of many of the findings from existing GWAS is that the most significant findings with the most robust statistical significance have only been associated with more extensive disease (stages III/IV). This may suggest genetic changes play a more important role in the aetiology of the disorder in this subset of women, but we cannot conclude this is the case without additional data from well phenotyped individuals with a stage I/II diagnosis.

A study using whole genome sequencing of members of an affected family with ovarian endometriosis highlights the power of this approach to identify novel mutations that might explain familial cases (Albertsen *et al.* 2019). The rapidly reducing cost of whole genome sequencing is likely to increase the use of this approach for analysis of at-risk families and could be one way to increase early diagnosis and better integrate GWAS data into diagnostic pathways. Another approach that shows promise involves analysis of levels of long non-coding RNAs or miRNAs in blood (Moustafa *et al.* 2020) or saliva (Balogova *et al.* 2022) which may also be useful in stratification of stages (Maier & Maier 2021).

Some useful insights that may accelerate new therapies have come from comparisons between endometriosis-associated SNPs and those associated with other traits and disorders. For example, a recent study linking risks of asthma and endometriosis (Adewuyi *et al.* 2022) is consistent with an important role for inflammatory processes that may be exacerbated by oestrogens in both conditions (Reyes-Garcia *et al.* 2021, Saunders & Horne 2021). In asthma, androgens can negatively regulate inflammation and Adewuyi and colleagues suggested that androgen receptor modulators might be explored as therapies for both conditions (Adewuyi *et al.* 2022). This suggestion is one that has been made in the context of endometriosis as a way of overcoming the negative side effects of the pain medication Danazol (Gibson *et al.* 2020); however, as our recent studies in mice demonstrate more studies on the impact of SARMs on endometirum are needed before they can be widely adopted in women (Simitsidellis *et al.* 2019).

In the case of migraine there is already discussion surrounding repurposing of drugs used to treat migraine for treatment of endometriosis-associated pain (Saunders & Horne 2021). Likewise reports that GWAS analysis of data sets related to depression and comparison to those of endometriosis identified a link to ‘gastric mucosa abnormality’ (Adewuyi *et al.* 2021) are consistent with new evidence that the gut–brain axis can play a role in pain pathways (Muller *et al.* 2020). These findings are likely to stimulate further studies on dietary modification as a non-drug therapy for both conditions.

The strong association between inflammation and endometriosis also means we can learn from new genomics-led approaches to identify targets and accelerate drug repurposing that have been applied to asthma and autoimmune disorders such as Sjogen’s syndrome (Fang *et al.* 2019). With data sets of priority targets now available (Fang & Knight 2022), comparisons using endometriosis datasets are a possibility and may yield new targets including those involved in crosstalk between inflammation and steroid signalling pathways.

## Conclusions and future perspectives

The endometrium is a tissue in which both steroids and inflammatory processes are implicated in normal function, and in disorders such as endometriosis, so the apparent association between genetic variants that have an impact on steroid receptor expression and/or steroid signalling and endometriosis risk appears to back up what we already know about the characteristics of the disorder (Saunders & Horne 2021). Challenges remain in linking changes in specific gene expression with causation and/or aetiology which is not surprising given the heterogeneity of the disease and the complex interrelationships between steroids (biosyntheisis/metabolism), steroid signalling pathways and changes in tissue function.

Montgomery and colleagues have argued that we may achieve additional breakthroughs in our understanding of the role(s) of gene mutations in the origins and pathogenesis of the disorder (Montgomery *et al.* 2020) by expanding our studies on somatic mutations in epithelial cells within the eutopic endometrium many of which may arise early in life (Lac *et al.* 2019): this is clearly an important area for future work.

Steroid regulation of the endometrium and endometriosis lesions which may contribute to risk and progression of the disorder will also be influenced by epigenetic changes to the genome and large-scale studies exploring DNA methylation data from women with endometriosis and comparisons to controls are now underway. These studies have expanded on those analysing the impact of methylation on expression of individual genes such as *ESR2* (Xue *et al.* 2007). Mortlock *et al.* analysed DNA methylation data from endometrium and blood samples of 66 women reporting genetic regulation of methylation in endometrium across the menstrual cycle that was not observed in blood and novel disease-related methylation quantitative trait loci including one near *GREB1*(Mortlock *et al.* 2019). Another study used stromal cells isolated from eutopic endometrium during the proliferative phase as well as* in vitro* cultures with E2 and/or progesterone in combination with analysis of the DNA methylome to compare epigenetic landscape and see if this was altered in endometriosis patients (Houshdaran *et al.* 2020). The authors reported finding pre-existing aberrant DNA methylation signatures in the cells from women with endometriosis and that these were not uniform throughout the patient group with those found in women with stage IV disease associated with a blunting of response to E2 treatment.

Another regulatory pathway that has been investigated in the context of hormone regulation of endometrium/endometriosis is that of non-coding RNAs (Vashisht *et al.* 2020). Whilst outside the scope of this review the miRNA field is a rapidly expanding one with some promising results linking miRNAs to disease mechanisms (Stejskalova *et al.* 2021). Further studies on epigenetic changes in the genome and non-coding RNA pathways are anticipated but they also need to be closely integrated with the insights from genomic studies.

In summary, the rapid explosion in the use of unbiased genomic approaches such as GWAS has led to a large body of data that consistently reports mutations in areas of the genome that appear associated with genes that regulate hormone-dependent gene expression (receptors, enzymes, transcription factors). These changes may explain some of the genetic risk associated with developing this disorder and other co-morbidities reported by patients. The next challenge is to integrate these data with changes in cell/tissue function and to use them as a platform for improvements in diagnosis, development of new therapies and care pathways.

## Declaration of interest

The author declares that there is no conflict of interest that could be perceived as prejudicing the impartiality of this review.

## Funding

Work in the author’s laboratory directed at understanding the role of sex steroids has been funded by the Medical Research Council http://dx.doi.org/10.13039/501100000265 UK (MR/N024524/1, G1100356/1).
